# A Case of Heatstroke Encephalopathy With Abnormal Signals on Brain Magnetic Resonance Imaging

**DOI:** 10.7759/cureus.17053

**Published:** 2021-08-10

**Authors:** Gentaro Hiramatsu, Masaki Hisamura, Makoto Murase, Yuriko Kukihara, Motohiro Nakamura

**Affiliations:** 1 Department of Critical and Emergency Medicine, Saitama Medical Center, Saitama Medical University, Kawagoe, JPN; 2 Department of Emergency Medicine, Saitama Medical Center, Saitama Medical University, Kawagoe, JPN

**Keywords:** heat stroke encephalopathy, magnetic resonance imaging, neurological, subcortex, paroxysmal sympathetic hyperactivity

## Abstract

Herein, we present a case of heatstroke encephalopathy with abnormal brain magnetic resonance imaging (MRI) signals. A 19-year-old man lost consciousness while working outdoors when the temperature was 35°C. His Glasgow Coma Scale score at presentation was E1V1M1, and his body temperature was 39°C. Chest computed tomography revealed bilateral infiltrates, and tests for urinary pneumococcal antigens were positive. He was diagnosed with heatstroke preceded by pneumococcal pneumonia. He was subsequently treated with antibiotics, and body surface cooling was performed. A diffusion-weighted brain MRI performed on day eight revealed abnormal bilateral hyperintensities from the cortex at the frontal lobe apex of the subcortex. Moreover, he had reduced spontaneity, dysarthria, nystagmus, tremor, and ataxia of both the upper limbs. He was diagnosed with heatstroke encephalopathy. On day 28 since admission, the abnormal MRI signals disappeared. Subsequently, the patient’s spontaneity improved, but his other neurological dysfunctions persisted. This case study demonstrates that MRI may not be a sensitive indicator of the prognosis of heatstroke encephalopathy.

## Introduction

Heatstroke encephalopathy presents with abnormal head magnetic resonance imaging (MRI) signals [1,2]. Ischemia-like lesions on MRI are caused by low blood flow and abnormal coagulation, whereas posterior reversible encephalopathy syndrome (PRES)-like lesions result from direct heat and angiogenic edema due to hypercytokinemia [3]. However, the use of this imaging method and its therapeutic consequences remain controversial. We herein discuss a case of heatstroke encephalopathy in which the patient presented with abnormal brain MRI signals.

## Case presentation

A 19-year-old man lost consciousness while working outdoors when the temperature was 35°C. He had a one-week history of cough and a three-day history of fever (body temperature, 37.6°C) before this episode. His medical history was otherwise unremarkable. He was rescued by an air helicopter. Moreover, he had a body temperature, blood pressure, heart rate, respiratory rate, oxygen saturation, Glasgow Coma Scale (GCS) score, and pupil diameter of 39°C, 62/29 mmHg, 152 beats/min (sinus rhythm), 36 breaths/minute, 88% (ambient air), E1V1M1, and 3.0/3.0 mm, respectively. Furthermore, the patient had a slow pupillary light reflex. An intravenous line was established, and tracheal intubation was performed. Thereafter, he was urgently transferred to our hospital. Upon admission, he had a body temperature, blood pressure, heart rate, oxygen saturation, GCS score, and pupil diameter of 41.7°C, 171/71 mmHg, 188 beats/min (sinus rhythm), 95% (intubated), E1V1M1, and 1.0/1.0 mm, respectively. Moreover, the pupillary light reflex was not observed. Laboratory investigations revealed increased creatine phosphokinase levels, thrombocytopenia, and elevated liver enzymes (Table 1).

**Table 1:** Laboratory findings of the patient on admission. P, phosphorus; ALB, albumin; CK, creatine kinase; AST, aspartate aminotransferase; ALT, alanine aminotransferase; LDH, lactate dehydrogenase; Cr, creatinine; BUN, blood urea nitrogen; Na, sodium; K, potassium; Cl, chlorine; T-Bil, total bilirubin; CRP, C-reactive protein; APTT, activated partial thromboplastin time; PT, prothrombin time; INR, International Normalized Ratio; WBC, white blood cell; RBC, red blood cell; Hb, hemoglobin; HCT, hematocrit; PLT, platelet.

Blood biochemistry	
P	7.3 g/dL
ALB	4.8 g/dL
CK	749 U/L ↑
AST	50 U/L ↑
ALT	43 U/L ↑
LDH	595 U/L ↑
Cr	1.67 mg/dL ↑
BUN	13 mg/dL
Na	135 mEq/L ↓
Cl	98 mEq/dL ↓
K	5.0 mEq/L ↑
T-Bil	1.0 mg/dL
CRP	0.42 mg/dL
Coagulation test	
APTT	23.5 sec ↓
PT	14.6 sec ↑
PT%	81%
PT-INR	1.14 ↑
D-dimer	2.15 μg/mL ↑
Complete blood count	
WBC	6,900/μL
RBC	4.86 million/μL
HGB	14.0 g/dL
HCT	41.6%
PLT	371,100/μL ↓

Computed tomography (CT) of the brain was unremarkable. A chest CT revealed bilateral infiltrates and atelectasis. The cerebrospinal fluid was tested to investigate the cause of his impaired consciousness, and the results ruled out meningitis. Tests for urinary pneumococcal antigens were positive, while those for human immunodeficiency virus antibodies were negative. He tested negative for coronavirus disease. Based on these findings, he was diagnosed with heatstroke, preceded by pneumococcal pneumonia. Subsequently, he received an infusion of chilled Ringer’s solution. Additionally, azithromycin, ceftriaxone, and vancomycin were administered.

**Figure 1:**
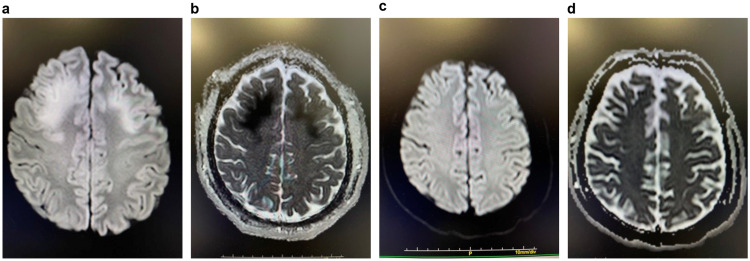
Magnetic resonance imaging findings on day eight. A. Brain MRI on day eight of hospitalization: diffused-weighted image showing abnormal signals from the cortex to the subcortical region of the bilateral frontal lobe apex. B. Brain MRI on day eight of hospitalization: the apparent diffusion coefficient map is low. C. Brain MRI on day 28 of hospitalization: previously noted abnormal signals from the cortex to the subcortical region of the bilateral frontal lobe apex have disappeared. D. Brain MRI on day 28 of hospitalization: the low signal of the apparent diffusion coefficient map has disappeared. MRI, magnetic resonance imaging.

The patient’s fever, chest infiltrates, and atelectasis gradually improved on day three. Blood cultures were unremarkable at baseline and on day four. The GCS score improved to E3VTM6 on day seven (without sedatives). Thus, the patient was extubated. Since his higher functions were impaired, a brain diffusion-weighted (DW)-MRI was performed on day eight (Figure 1).

**Figure 2:**
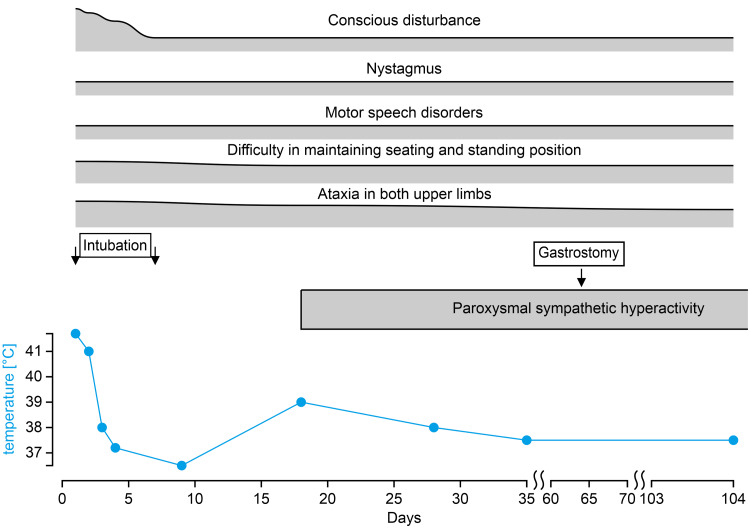
The clinical course of the patient.

Abnormal signals were noted from the cortex at the frontal lobe apex on both sides of the subcortex. The apparent diffusion coefficient map showed low signals, but arterial spin labeling (ASL) revealed no blood flow abnormalities. Reactive eye movements and occasional indistinct vocalization were observed. The pupils were circular and measured 3 mm in diameter. The pupillary light reflex was intact, but nystagmus was observed. Moreover, tremors in the limbs and ataxia were noted. Epilepsy was ruled out after electroencephalography. On day 18, sympathetic hypertonia with fever (body temperature, 39°C), paroxysmal tachypnea (respiratory rate, 30 breaths/minute), and tachycardia (heart rate, 140 beats/min) were noted. The patient was diagnosed with paroxysmal sympathetic hyperactivity (PSH), and dexmedetomidine was administered. The sympathomimetic symptoms were alleviated on day 28 using gabapentin. Brain MRI performed on day 28 after admission revealed the disappearance of the abnormal signals. Thereafter, dexmedetomidine was discontinued on day 35. Although the patient’s spontaneity improved, his other neurological symptoms and PSH persisted. Due to poor swallowing, a gastrostomy was performed on day 64, and the patient was transferred to another hospital for rehabilitation on day 104 (Figure 2).

At the time of transfer, the patient was able to provide simple answers. The patient was still unable to sit or stand on his own owing to muscle weakness caused by prolonged bed rest. We have received no information from the transfer location regarding the patient’s disposition.

## Discussion

Brain tumors, cerebrovascular accidents, infectious diseases, intoxication/metabolic diseases, autoimmune/inflammatory diseases, cerebral edema, and hydrocephalus have similar presentations on MRI [4]. In our patient, the abnormal brain MRI signals disappeared without obtaining any specific treatment, and the ASL did not indicate a decline in cerebral blood flow. Therefore, a vascular lesion was ruled out, and cerebral edema was suspected. The frontal lobe function was not evaluated in our patient. However, his condition improved in terms of spontaneity and MRI findings without the need for sedatives. Thus, the reduced spontaneity was likely related to the abnormal MRI findings.

The patient exhibited other neurological symptoms. Nystagmus and ataxia suggested cerebellar abnormalities, but there were no corroborative MRI findings. This absence of findings was likely related to the timing of the MRI. In two previously reported cases, abnormal MRI signals disappeared within a week [5] and a month [6], respectively. A previous report of a patient diagnosed with cerebellar atrophy, but without neurological sequelae for two years, demonstrated that the abnormal MRI findings in both the frontal and parietal lobes disappeared after a month [7]. In another case, the brain DW-MRI revealed abnormal signals in the frontal, temporal, and occipital lobes, with cerebellar ataxia after two months [1]. In another report, abnormal MRI findings were not detected during the early stages of the disease and were only found on day two after hospitalization [1]. In our patient, a brain MRI was performed on day eight. However, it was unclear whether the abnormal signals, other than those in the frontal lobe, indicative of clinical neurological signs, disappeared once the heatstroke ceased or whether they remained unidentified.

The cerebellum is sensitive to heat [2]. The Purkinje cells in the cerebellum are sensitive to fever-induced damage [7]. A previous report has described cerebellar ataxia due to high fever secondary to malignancy [8]. In another report, the Purkinje cells were selectively depleted in patients who died of high fever due to serotonin syndrome [8]. The cerebellum contains several heat shock proteins that could be involved in the pathogenesis of heatstroke encephalopathy [9].

Although the number of reports describing abnormal head MRI signals in patients with heatstroke encephalopathies is increasing, MRI findings can only corroborate the diagnosed neurological symptoms. Additionally, there is no consensus on the optimal timing for performing a brain MRI for these patients [5,8]. The sensitivity of brain MRI in detecting abnormal neurological findings was low in patients with heatstroke. Brain MRI findings of patients with heatstroke did not affect the treatment protocol and had a low clinical value. Neurological signs detected clinically were more relevant because it was impractical to increase imaging frequency to obtain positive results.

## Conclusions

The number of abnormal MRI findings in patients with heatstroke encephalopathy has increased over the years. Although MRI has been recommended to guide the treatment plan for heatstroke encephalopathy, its sensitivity is low owing to the lack of consensus regarding its timing. Moreover, in this case, the images were nonspecific and did not explain the neurological symptoms. Thus, heatstroke encephalopathy should be diagnosed and treated clinically, regardless of the MRI findings. The fact that abnormal MRI signals are obtained in the brain in heatstroke encephalopathy may be of academic value, but in clinical practice, it should be used only as a reference. Moreover, the diagnosis and treatment should be based on the patient’s clinical symptoms.
